# Assessing the vibration perception threshold in a community sample of adult Ghanaians

**DOI:** 10.1371/journal.pone.0291606

**Published:** 2023-11-08

**Authors:** Francis Tanam Djankpa, Albert G. B. Amoah, Festus Komla Adzaku, Eric Aidoo, Isaac Boateng

**Affiliations:** 1 Department of Physiology, School of Medical Sciences, College of Health and Allied Sciences, University of Cape Coast, Cape Coast, Ghana; 2 National Diabetes Management and Research Centre, Korle-Bu Teaching Hospital, Korle Bu, Accra, Ghana; 3 Department of Medicine and Therapeutics, School of Medicine and Dentistry, College of Health Sciences, University of Ghana, Accra, Ghana; 4 Department of Physiology, Family Health Medical School, Family Health University College, Accra, Ghana; 5 Department of Anatomy, School of Medical Sciences, College of Health and Allied Sciences, University of Cape Coast, Cape Coast, Ghana; Muhimbili University of Health and Allied Sciences School of Medicine, UNITED REPUBLIC OF TANZANIA

## Abstract

**Background:**

The vibration perception threshold (VPT) helps evaluate human somatosensory function and diagnose peripheral neuropathy. To optimize its use as a primary neurologic tool, it is imperative to establish its typical values in healthy subjects and assess the factors affecting its variability in an individual to ensure consistency in its application.

**Methods:**

Demographic data and a brief medical history were collected from 391 non-diabetic adults aged 30–80 at Kpone-on-Sea in Ghana. The VPT was measured at the tip of the big toe, the medial malleolus, the tip of the middle finger, and the head of the ulna of each participant using a Horwell Neurothesiometer. The variability of VPT was assessed vis-à-vis the following factors: gender, age, fasting plasma sugar and body mass index.

**Results:**

The mean age of participants was 48.4 ± 0.7 years, and the female-to-male ratio was 1.46. The overall VPT values ranged from 5.74 ± 0.14 volts to 8.55 ± 0.18 volts in the lower limbs and 3.61 ± 0.06 volts to 5.00 ± 0.08 volts in the upper limbs. Age was found to be the only factor that could predict VPT for both the lower and upper limbs (*P* < 0.001). One-Way Analysis of Variance with Tukey’s posthoc showed that the VPT in the feet was significantly higher than that in the hands.

**Conclusions:**

Generally, the VPT was high on proximal sites and low on distal sites indicating that the vibration sensation increased from proximal to distal direction. Therefore, distal areas should be used for VPT testing with a Neurothesiomer. Age was found to be the only factor that affected VPT variability. Hence, the practical application of VPT will require age-specific reference ranges to cater for older adults.

## Background

The human nervous system, consisting of the central and peripheral divisions, is a complex network of pathways that allows humans to interact successfully with their environment and maintain homeostasis [1]. The central nervous system comprises the brain and spinal cord, whereas the peripheral nervous system consists of 12 pairs cranial nerves and 31 pairs of spinal nerves [2]. Every peripheral nerve performs a specialized function in a specific body part which may be compromised when the nerve is damaged. The peripheral nervous system can be damaged by numerous disease processes resulting in peripheral neuropathy [3]. Usually, the clinical diagnosis of peripheral neuropathy in Ghana is by qualitative assessment using a tuning fork in combination with clinical examination and history taking. Although quick and straightforward for gross evaluations, using a tuning fork is unsatisfactory for measuring thresholds at which vibration becomes perceptible because it has no calibration and cannot be quantified [4]. The qualitative method of assessing peripheral neuropathy using a tuning fork is highly subjective, unreliable and may present results with considerable inter and intra-examiner variations [4].

As a way of overcoming the variations in the qualitative assessment of neuropathy using a tuning fork, new methods have emerged. One such method is the use of a neurothesiometer. A neurothesiometer is a battery-powered device used to quantify the vibration perception threshold (VPT) accurately. The VPT is the least amount of vibration that can be perceived. In a physiological state of normal sensory function, the vibration perceived by receptors differs from that perceived in a pathologic nerve damage condition. This differential sensation of vibration forms the basic principle guiding neurothesiometry. Hence, in clinical practice and research, a threshold is often established after trials in healthy individuals and used as a standard for diagnosing possible neurologic deficits in patients. Neurothesiometry is a clinically validated tool for rapid screening, early detection, and longitudinal evaluation of persons at risk of sensory deficits associated with many diseases and conditions [5]. It is a quick, simple, and non-invasive quantitative method of assessing sensory deficits. Using a neurothesiometer to diagnose peripheral neuropathy adds more meaning to clinical findings made during physical examination. It enables grading in the assessment, comparison among patients and precision in monitoring the progress of neurologic disorders during treatment.

The vibration perception threshold (VPT) is of great medical importance. It is a fundamental tool for evaluating human somatosensory function and diagnosing peripheral neuropathy in clinical practice and research, especially in diabetic patients [6, 7]. It can be used as a tool for research into occupational health to screen workers at workplace for peripheral neuropathy caused by direct vibration or toxic substances [7, 8]. The VPT is an effective predictor of the risk of trophic ulceration of the feet and risk of amputation in diabetic patients and, therefore can be used to target foot-care education as a preventive measure [9–13]. The VPT is also widely applied in community-based research, occupational health screening in industries and ambulatory clinics [5].

Although several studies have been conducted to determine the VPT in other populations, these findings cannot be applied to the Ghanaian population because VPT values are influenced by age, race, gender, occupation, site of measurement, and the type of instrument used. Typically, Nolte and colleagues measured the VPT of non-diabetic subjects at the big toe in Netherlands using a biothesiometer and found converted absolute values ranging from 0.79 (in subjects less than 39 years) to 5.74 (in subjects aged above 190 years) [13]. At other body sites, the VPT values were as follows: 0.94 to 4.31 at the insteps of the foot, 0.83 to 4.68 at the lateral malleolus and 0.30 to 0.52 at the wrist, with the same age range as in the big toe [13]. Similarly, Ghosal and colleagues measured the VPT at the big toes and the medial malleoli among non-diabetic Indians aged 20–65 years using a biothesiometer. They found the average value to be 11.3 ± 4.9 mV [17]. In addition, Williams and Colleagues also measured the VPT among non-diabetic individuals in the UK and found the values adjusted for age to be 1.0623 V, 1.0517 V, 1.1727 V, 1,1586 V at the left big toe, right big toe, left malleolus and right malleolus respectively [14]. Obviously, the VPT values vary widely depending on the population dynamics (anthropometric, sociodemographic factors), the site of the test, the nature of the instrument/tool used and other confounding factors. The VPT is important and clinically relevant in assessing peripheral neuropathy among diabetic patients. Hence, there is a need for every country or sub-region to establish normative/normal values, which will serve as a reference range for diagnosing peripheral neuropathy caused by diabetes and other conditions [11].

Considering the medical importance of the use of VPT in the assessment of peripheral nerve function, there needs to be baseline information on VPT in Ghana and information on determinants and variability of VPT among Ghanaians. Therefore, there is a need to assess the usefulness of the VPT as a primary neurologic tool in investigating peripheral nerve function for clinical application in Ghana. This study aims to establish the typical VPT values in healthy adult Ghanaians using a Neurothesiometer and to assess the factors affecting its variability. “Healthy subjects” as used in this work refers to non-diabetic individuals who met the inclusion criteria spelt out in the methodology section. Our findings will be helpful in the clinical application of VPT in diagnosing peripheral neuropathy caused by diabetes, workplace-related neurotoxicity, and other conditions. Furthermore, based on our findings, further research can be conducted to assess the potential of using the VPT values to predict the risk of trophic foot ulceration and amputation among diabetic patients.

## Methods

### Study population

This research was a community based cross-sectional study carried out at Kpone-on-Sea near Tema in the Greater Accra Region of Ghana. The research protocol was reviewed and approved by the Ethics and Review Board of the University of Ghana School of Medicine and Dentistry. An informed written consent was obtained from all the subjects who willingly took part in this research.

### Inclusion criteria

Individuals on medication for diabetes, high blood pressure and all forms of neurologic deficits/disorders were excluded from this study. During the screening, other individuals who were diagnosed as having diabetes (using the fasting plasma glucose combined with the glucose tolerance tests) were equally excluded from the studies. On arrival, subjects were made to complete the personal and demographic data section of the WHO STEPS questionnaire. The STEPS questionnaire is a data collection tool in research that was designed by the World Health Organization (WHO) in 2002 to obtain core data on the established risk factors that determine major disease burden by gathering demographic and behavioral information from participants [15, 16]. In some cases, however, individuals (with no history of neurological deficit) found to have abnormally high VPT values compared to others were equally excluded from this study.

### Measurement of the VPT

The VPT (in volts) was measured among 391 healthy subjects aged 30–80 years using a neurothesiometer (Horwell Scientific Laboratory Supply Limited, Wilford Industrial Estate, Wilford, Nottingham, UK). The following sites on the limbs were chosen for measurement as previously used by other researchers [13, 14, 17–19]: the tip of big toe and the medial malleolus (on the feet) and the tip of middle finger and the head of ulna (on the hands). By convention, the following nomenclature was used: V1 (VPT at the left tip of big toe), V2 (VPT at the left medial malleolus), V3 (VPT at the left tip of middle finger), V4 (VPT at the left head of ulna), V5 (VPT at the right tip of big toe), V6 (VPT at the right medial malleolus), V7 (VPT at the right tip of middle finger and V8 (VPT at the right head of ulna). The vibrating probe of the neurothesiometer was placed firmly over the testing sites at about 90°C to the skin such that its weight rested fully on the test site. The voltage was increased gradually from 0.00 V at a rate of about 0.5 V per second. Subjects were instructed to respond, “yes” as soon as they felt the vibration sensation as previously described [20, 21]. After receiving a pre-test on the forehead, the VPT was then measured on the designated sites.

### Measurement of other parameters

The blood pressure (systolic and diastolic) of subjects was measured three (3) times in the right arm at five (5) minutes interval using a digital automated blood pressure monitor, Model No: HEM-907XL by Omron Health Care Inc. The mean of the last two measurements was used. Subjects then had a venepuncture on a forearm vein with a butterfly needle and 2.5 ml of venous blood was drawn and stored in fluoridated EDTA tubes. The fluoridated blood samples were kept on ice and centrifuged at a speed of 3000 rpm within 15 minutes after sampling using a bench top centrifuge (Model DURAFUGE 100, 02–2004 by JOUAN Industries SAS). The resultant plasma was stored in Eppendorf tubes at -80°C in the Diabetes Research Laboratory of the Korle-Bu Teaching Hospital and analysed thereafter. The plasma samples were analysed to determine the fasting plasma glucose (FPG) in duplicate using glucose oxidase kits and normal and elevated precision multi-sera controls (Randox Laboratories, County Antrum, UK) on an Erba Smartlab Automatic Chemistry Analyzer (Erba, TransAsia). A glucose tolerance test was equally performed and used in combination with the fasting plasma glucose to diagnose individuals with diabetes. The height and weight were measured in duplicate and the mean was found. These measurements were taken with a heavy-duty electronic Seca 770 floor digital scale (Seca, Hamburg, Germany). The Body Mass Index (BMI) of each subject was computed using the 1995 WHO standard from the weight and height as: BMI = weight (Kg) / height^2^ (m^2^).

### Statistical analysis

The field data was entered into Microsoft Excel spreadsheet. Statistical analysis of the result was performed using SPSS package version 26. Descriptive statistics were presented as mean ± SEM (standard error of mean). Inferential analysis was carried out at 95% confidence interval and values were considered significant at *P* < 0.05. Pearson’s correlation and Chi-Square test were used to find the association between VPT and other factors and multiple regression analysis was used to determine the predictors of VPT. One-way ANOVA with the Tukey’s post hoc multiple comparison test was used to compare site variability of VPT.

## Results

The distribution of 391 participants by age group is shown in **Table 1**. The mean age was 48.4 ± 0.7 years. The female to male ratio was 1.46 and the modal age range was 30–39 years. The least represented age range was 50–59 years **(Table 1)**. The BMI of the females was significantly higher as compared to that of the males (*student’s t-test*, p < 0.01) **(Table 1)**. Moreover, 25.1% of the sample population were found to be obese **(Table 1)**.

**Table 1:** Age and body mass index (BMI) distribution of participants by gender.

		Distribution by Gender		
		Male	Female	Overall
**Age Group**	30–39 years	35.8%	29.3%	32.0%
	40–49 years	24.5%	26.3%	25.6%
	50–59 years	18.9%	15.0%	18.9%
	60–80 years	20.8%	25.4%	23.5%
**Obesity**	Yes	19.4%	80.6%	25.1%
	No	47.8%	52.2%	74.9%
**BMI**		24.5 ±0.32**	28.00 ±0.33**	26.56 ±0.23

Mean Age: 48.4 ± 0.7 years

*: *P* -value <0.05

**: *P* -value <0.01

***: *P* -value <0.001)

### Site variability of the VPT on the left limbs within and across the age groups

Comparing the VPT across the various age groups, there was a significant difference between the VPT at V1 (tip of left big toe) across the age groups, ANOVA: F (3, 387) = 98.46, *P* < 0.01 **(Table 2)**. Tukey’s post hoc multiple comparison revealed that the VPT at V1 for age groups 30–39 years, 40–49 years, 50–59 years, and 60–80 years were significantly different from one another, *P* <0.01 **(Table 2)**. The VPT values at V2 (left medial malleolus) were significantly different across the age groups, ANOVA: F (3, 387) = 47.2, *P* < 0.01 **(Table 2)**. Tukey’s post hoc multiple comparison shows that V2 values for age groups 30–39 years, 40–49 years, 50–59 years, and 60–80 years were significantly different from one another, *P* <0.01 **(Table 2)**. There was a significant difference between VPT values at V3 (tip of left middle finger) across the age groups, ANOVA: F (3, 387) = 13.62, *P* < 0.01 **(Table 2)**. Tukey’s post hoc multiple comparison shows no significant difference between V3 values for age groups 30–39 years, 40–49 years and 50–59 years but V3 values for age group 60–80 years were significantly higher than those of the other age groups **(Table 2)**. The VPT values at V4 (head of left ulna) were significantly different across the age groups ANOVA F (3, 387) = 7.80, *P* < 0.01 **(Table 2)**. Tukey’s post hoc multiple comparison further revealed that V4 values were significantly higher for age group 60–80 years compared to the other age groups, which were not significantly different from one another **(Table 2)**. Comparison of the overall VPT values indicate there is a significant difference across the testing sites on the left limbs, ANOVA: F (3, 387) = 98.5, p < 0.0001. Tukey’s post hoc multiple comparison showed that the mean VPT value at the tip of left middle finger (V3) was significantly lower than that at the head of left ulna (V4), which was also significantly lower than that at the tip of left big toe (V1). The mean VPT at the left medial malleolus (V2) was significantly highest among all the test sites on the left limbs **(Table 2)**.

**Table 2:** Comparison of VPT in the left limbs within and across the age groups.

Mean VPT(±SEM)/volts				
Age groups	V1	V2	V3	V4
**30–39 years**	4.00***±0.13	6.50** ±0.19	3.22 ±0.09	3.59 ±0.12
**40–49 years**	5.17*** ±0.17	7.83** ±0.29	3.57 ±0.12	3.85 ±0.14
**50–59 years**	5.71*** ±0.20	8.61*** ±0.33	3.52 ±0.0.13	3.72 ±0.15
**60–80 years**	8.81*** ±0.33	11.35*** ±0.14	4.28*** ±0.20	5.60*** ±0.17
**Overall**	5.76*** ±0.14	8.38*** ±0.18	3.61*** ±0.06	3.92*** ±0.07
**p-values**	0.000	0.000	0.000	0.000

SEM: Standard Error of Mean; VPT: Vibration Perception Threshold; V1: tip of left big toe, V2: left medial malleolus, V3: tip of left middle finger, V4: head of left ulna

*: *P* -value <0.05

**: *P* -value <0.01

***: *P* -value <0.001).

### Site variability of the VPT on the right limbs within and across the age groups

Table 3 shows the comparison of VPT values on the right limbs across the various age groups. There was a significant difference between VPT values at V5 (tip of right big toe) across the age groups ANOVA F (3, 387) = 47.2, *P* < 0.01 **(Table 3)**. Tukey’s post hoc multiple comparison reveals that the V5 values for age groups 30–39 years and 40–49 years were not significantly different from each other. The V5 values for age group 50–59 years were significantly higher than those of age groups 30–39 and 40–49 years. The V5 values for age group 60–80 years were significantly higher than those for age group 50–59 years. The VPT values at the right medial malleolus (V6) were significantly different across the age groups, ANOVA F (3, 387) = 38.8, *P* < 0.01. Tukey’s post hoc multiple comparison shows that V6 values for age group 60–80 years were significantly higher than those for age group 50–59 years, which were also significantly higher than the reaming age groups. There was no significant difference between V6 values for age groups 30–39 and 40–49 years **(Table 3)**. The VPT values at V7 (tip of right middle finger) were significantly higher for the age group 60–80 years as compared to the other age groups (30–39, 40–49, and 50–59 years), which did not show any significant difference compared to one another, ANOVA F (3, 387) = 9.5, *P* < 0.01 with Tukey’s post hoc multiple comparison test **(Table 3)**. Finally, the variation in VPT values at V8 (head of right ulna) had a different pattern **(Table 3)**. The values for the 60–80 years age group were significantly higher than those of the other age groups. Whereas the values for age group 30–39 years were significantly the lowest among all. The VPT for age groups 40–49 and 50–59 years were significantly not different from each other, ANOVA F (3, 387) = 11.9, *P* < 0.01 with Tukey’s post hoc multiple comparison test **(Table 3)**. Comparison of the overall VPT values indicate that there was a significant difference across the testing sites on the right limbs similar to the left limbs, ANOVA: F (3, 387) = 47.2, p < 0.0001. Tukey’s post hoc multiple comparison showed that the mean VPT value at the tip of right middle finger (V7) was significantly lower than that at the head of right ulna (V8), which was also significantly lower than that at the tip of right big toe (V5). The mean VPT at the right medial malleolus (V6) was significantly highest among all the test sites on the right limbs **(Table 3)**.

**Table 3:** Comparison of VPT in the right limbs within and across the age groups.

Mean VPT(±SEM)/volts				
Age groups	V5	V6	V7	V8
**30–39 years**	4.41 ±0.17	6.83 ±0.22	3.00 ±0.13	4.61** ±0.1072
**40–49 years**	5.19 ±0.20	8.14 ±0.30	3.85 ±0.15	4.78 ±0.14
**50–59 years**	5.82*** ±0.24	8.39*** ±0.35	3.72 ±0.15	4.89 ±0.15
**60–80 years**	8.08*** ±0.33	11.47*** ±0.43	4.61*** ±0.16	5.78*** ±0.21
**Overall**	5.74*** ±0.14	8.55*** ±0.18	3.92*** ±0.08	5.00*** ±0.08
p-values	0.00	0.00	0.00	0.00

SEM: Standard Error of Mean; VPT: Vibration Perception Threshold; V5: tip of right big toe, V6: right medial malleolus, V7: tip of right middle finger, V8: head of right ulna

**: *P* -value < 0.01

***: *P* -value <0.001.

### Site variability of the VPT between males and females

Comparing the VPT between males and females, there was no significant difference of mean VPT values of the left and right limbs between males and females at all sites, *P* > 0.05 (Tables 4 and 5).

**Table 4:** Comparison of VPT in the left limbs between males and females.

Mean VPT(±SEM) volts in the Left Limbs by Gender				
Gender	V1	V2	V3	V4
**Male**	5.58 ±0.22	7.99 ±0.25	3.57 ±0.10	5.00 ±0.12
**Female**	5.88 ±0.18	8.64 ±0.25	3.65 ±0.08	5.00 ±0.10
**Overall**	5.76 ±.14	8.38±0.18	3.6 ±0.06	4.99±0.07
p-values	0.28	0.07	0.56	0.87

SEM: Standard Error of Mean; VPT: Vibration Perception Threshold; V1: tip of left big toe, V2: left medial malleolus, V3: tip of left middle finger, V4: head of left ulna.

**Table 5:** Comparison of VPT in the right limbs between males and females.

Mean VPT(±SEM) volts in the Right Limbs by Gender					
Gender		V5	V6	V7	V8
**Male**		5.52 ±0.21	8.24 ±0.30	3.82 ±0.12	4.83 ±0.11
**Female**		5.88 ±0.18	8.76 ±0.24	4.00 ±0.10	5.09 ±0.11
**Overall**	**Mean VPT**	5.74 ±0.14	8.55±0.18	3.92 ±0.08	5.00 ±0.08
	*P* **-value**	0.20	0.16	0.24	0.12

SEM: Standard Error of Mean; VPT: Vibration Perception Threshold; V5: tip of right big Toe, V6: right medial malleolus, V7: tip of right middle finger, V8: head of right ulna.

### Site variability of the VPT on the left and right limbs

Comparison of VPT values between the corresponding points on the left and right limbs indicates that the VPT on the tip of left middle finger (V3) was significantly lower than that on the tip of the right middle finger (V7), (paired *t-test*, *P* < 0.001), meaning V3 was more sensitive to vibration than V7 **(Table 6)**. The VPT values at all the remaining corresponding sites (namely V1 versus V5, V2 versus V6 and V4 versus V8) did not show any significant different from each other (paired *t-test*, p > 0.05).

**Table 6:** Paired samples *t-test* of the difference in the VPT at various points for both right and left limbs.

Paired Samples Test									
		Paired Differences					t	df	Sig. (2-tailed)
		Mean difference	Std. Deviation	Std. Error Mean	95% Confidence Interval of the Difference				
					Lower	Upper			
Pair 1	V1—V5	0.02	2.18	0.11	-0.20	0.24	0.17	390	0.86
Pair 2	V2 –V6	-0.17	2.92	0.15	-0.46	0.12	-1.14	390	0.25
Pair 3	V3—V7	-0.31	1.34	0.07	-0.44	-0.18	-4.57	390	0.000
Pair 4	V4 –V8	0.01	1.42	0.07	-0.13	0.16	0.20	390	0.84

df: degree of freedom; V1: Tip of left Big Toe, V5: Tip of right Big Toe, V2: left Medial Malleolus, V6: right Medial Malleolus, V3: Tip of left Middle Finger, V7: Tip of right Middle Finger V4: Head of left Ulna, V8: Head of right Ulna.

### Factors affecting the VPT variability

Correlation analysis revealed that fasting plasma glucose, and BM1 had no significant correlation with VPT of the left and right limbs (p>0.05) **(Table 7)**.

**Table 7:** Relationship between baseline characteristics and VPT of the left and right limbs.

		Left Limbs		Right Limbs	
Site of Testing	Model	Pearson coefficient	*p*-value	Pearson coefficient	*p*-value
V1	FPG	-0.03	0.54	-0.07	0.19
	BMI	-0.01	0.86	0.05	0.31
V2	FPG	-0.08	0.13	-0.04	0.50
	BMI	0.08	0.13	0.02	0.68
V3	FPG	-0.04	0.21	-0.09	0.09
	BMI	-0.01	0.41	-0.04	0.42
V4	FPG	-0.02	0.75	-0.05	0.30
	BMI	0.02	0.75	0.02	0.76
	**Model**	**Pearson Chi-Square**	**p-value**	**Pearson Chi-Square**	**p-value**
V1	Age group	117.4	0.001	121.4	0.015
	Sex	23.0	0.575	29.2	0.509
	Hypertension	16.2	0.909	23.1	0.809
	Alcohol intake	26.0	0.410	31.7	0.381
V2	Age group	96.9	0.370	111.0	0.098
	Sex	35.1	0.281	28.3	0.607
	Hypertension	41.6	0.097	29.2	0.559
	Alcohol intake	27.3	0.659	32.7	0.382
V3	Age group	328.6	0.000	282.1	0.000
	Sex	55.6	0.241	50.3	0.458
	Hypertension	50.2	0.424	65.0	0.075
	Alcohol intake	52.4	0.345	62.9	0.104
V4	Age group	279.6	0.000	254.0	0.003
	Sex	71.3	0.173	53.6	0.843
	Hypertension	61.9	0.445	91.9	0.016
	Alcohol intake	75.9	0.095	62.7	0.559

SBP: Systolic blood pressure, DBP: Diastolic blood pressure, BMI: Body Mass Index; FBG: Fasting plasma glucose, V1: tip of left big toe, V2: left medial malleolus, V3: tip of left middle finger, V4: head of left ulna

Moreover, a chi-square test revealed a significant association between the age groups of the participants and the VPT of the tip of middle finger, tip of big toe, and medial malleolus (p<0.05). However, the VPT of the head of ulna for both left and right limbs had no significant association with the age of participants **(Table 7)**.

## Discussion

The primary aims of this study were to establish the VPT at different points and to assess the factors that may affect its variability. The study established the measurement of VPT among Ghanaian adults, marking the onset of an exploration of the potential application of neurothesiometry as a basic tool for peripheral nerve function assessment in Ghana. Here, we defined the use of the big toe, the medial malleolus, the tip of the middle finger and the head of ulna as testing sites in the measurement of VPT [18].

Various methods have been used to assess peripheral nerve function. These include clinical examination (coupled with the use of tuning fork), electromyography, nerve conduction test and quantitative sensory testing [22]. Clinical examination, is quick but non-quantitative and highly subjective [23]. Electromyography and nerve conduction tests are the gold standard but they are expensive and time consuming. In addition, they can be invasive and require sophisticated equipment and high skilled technicians for their application [24]. Quantitative sensory testing on the other hand is non-invasive, can be carried out quickly using less expensive equipment (e.g. neurothesiometer, biothesiometer) by an assessor with minimal training [25]. However, being a hand-held instrument, the use of a neurothesiometer presents with the following limitation: difficulty in maintaining a constant rate of increase of stimulus intensity over a long period of time and difficulty in maintaining the probe over the testing site [26].

The use of a neurothesiometer as a quantitative sensory testing to evaluate vibration sensation, has the potential for a wider application in medicine, diabetic clinics and community based research as compared to the gold standard. When established, neurothesiometers could easily be made available in hospitals in Ghana as basic neurologic tools in the diagnosis of peripheral neuropathy. The choice of body site tested in this research was based on literature on one hand, and deliberate on the other hand. Being a maiden research in Ghana, it is essential to maximize the body sites tested in order to pave a way for subsequent research to narrow down to fewer sites. Hence our choice of eight sites in each individual. The extent to which the VPT varies at different sites in an individual and the variation from one individual to another is important in establishing normal range to serve as standards in the assessment of peripheral nerve function.

### VPT in the upper and lower limbs

In this study, VPT values measured on the left and right feet were significantly higher (*P* <0.001) than that measured on the left and right hands indicating that the hands are more sensitive to vibration than the feet **(Tables 3 and 4)**. The observed high sensitivity to vibration in the hands can be attributable to the cortical representation of body areas on the sensory homunculus, which suggests that body areas with larger cortical representation are more sensitive. The hands have larger cortical representation as compared to the feet, justifying the observable higher sensitivity within the hands as observed in a study by Martin and colleagues [27].

### Site variability of VPT

Within each limb, the VPT measured at the medial malleolus was significantly higher than that at the tip of the big toe, which suggests that the tip of the big toe is more sensitive to vibration than the medial malleolus. Similarly, the VPT at the tip of the middle finger was lower than that at the head of ulna, indicating that the tip of middle finger is more sensitive to vibration than the head of ulna. This could be explained by the fact that extremities have a greater representation on the homunculus than the proximal parts. Generally, body parts that have a larger representation on the homunculus are more sensitive to tactile stimulation. Similarly, body parts with higher receptor density have smaller receptive fields and are more sensitive to mechanical stimulation, which includes vibration sensation [28–32]. Duncan and Boynton in their work titled “Tactile Hyperacuity Thresholds Correlate with Finger Maps in Primary Somatosensory Cortex (S1)”, reported that the tactile hyperacuity is largely represented by the fingers in S1 [33]. However, in a study to evaluate the VPT and the law of mobility in both the upper and lower extremities of diabetes mellitus patients, Manivannan and colleagues, found results which were not consistent with the findings of this present study. They reported that VPT values at the distal areas were higher than the proximal areas of the foot [34]. In line with our findings, Gu and Griffin, in their work to investigate the spatial summation of vibrotactile sensations at the foot, reported that the vibrotactile threshold decreased in the hands compared to the feet [35]. Similarly, Stuart and others also found that the fingertips were most sensitive to vibration as compared to other body parts tested in their study [36]. As mentioned earlier, the VPT measured at the head of ulna in the present study was also significantly higher than that measured at the tip of middle finger indicating that the tip of the middle finger is more sensitive to vibration than the head of the ulna **(Tables 2 and 3)**. Similar to our findings, Stuart and colleagues., in their study on the effect of aging on VPT at various regions, found the fingertip to be the most sensitive region for detection of vibrations in majority of the subjects [36]. This can be justified with the receptive field, the number of receptors and tactile acuity of the fingertip. The tip of fingers have higher density of receptors, smaller receptive fields and higher tactile acuity [11, 12] justifying why they are more sensitive to vibration than the head of ulna.

A paired sample *t-test* of the difference in VPT at the various testing sites for both right and left limbs shows that the tip of the left middle finger is significantly lower (*P* <0.001) than the tip of the right middle finger **(Table 6)**. In a similar study on the vibrotactile threshold measurement for detecting peripheral neuropathy, Duke and others., found a statistically higher vibration thresholds on the right arm and leg as compared to the left limbs [21]. These findings could be attributed to the general higher prevalence of right-handedness in the population compared to the left-handedness, where the right hand is often used for activities. The frequent use of the right hand more than the left could probably lead to some adaptation by mechanoreceptors to vibration, leading to reduced sensation of vibration by the mechanism of habituation [37, 38]. Since the prevalence of handedness was not assessed in our study population, this finding is not well understood and requires further studies. Meanwhile, the tip of the right and left big toes (left and right) were not significantly different from each other as was the case in the left and right medial malleolus and the head of left and right ulna **(Table 6)**.

Results from the correlation and chi-square test analysis in the present study suggest that age is the only factor that significantly influences the VPT among other factors like gender, BMI, FPG, alcohol intake and blood pressure **(Table 7)** [4, 15, 16, 21, 39, 40]. In our study, age was found to have a significant positive correlation (p<0.001) with VPT values at all the four testing sites on both right and left limbs as has been reported in several other studies conducted in healthy subject [21, 36, 39, 40]. The VPT values increase with increasing age meaning the vibration sensation decreases with increasing age. This agrees with another study conducted among adults in good health in Australia by Stuart and others [36].

Our findings further showed that females had a significantly higher BMI (*P* < 0.05) than males **(Table 1)**. In a cross sectional study conducted among adults in Sri Lanka to estimate the relation between BMI and body fat percentage in relation to age and gender, Ranasinghe and colleagues found a significant effect of age and gender on the BMI of the study population with females having a higher BMI than males similar to our study [40].

## Conclusions

Site variability of VPT revealed that the tip of big toe for both left and right limbs were more sensitive to vibration than the left and right medial malleolus. Similarly, the tip of middle finger for both the right and left limbs were more sensitive to vibration than the head of right and left ulna. Generally, the lower limbs had increased VPT values than the upper limbs indicating that the upper limbs were more sensitive to vibration. Moreover, the VPT decreased from proximal to distal direction indicating that vibration sensitivity increased in the same direction. The distal areas (tip of middle finger and tip of big toe) are therefore more sensitive to vibration and should be used for VPT testing. The effect of age on the variability of VPT indicates that loss of sensitivity with ageing was more severe in the feet than in the hands. VPT testing for the purpose of diagnosing peripheral neuropathy in older population will therefore be more reliable at distal sites of the hands as compared to the feet. Our results show that age is the most important factor affecting VPT variability and the VPT measured on the feet is significantly higher than that measured on the hands. These findings will serve as baseline for the Ghanaian population and could lead to the establishment of reference values for the VPT as a clinical tool in the diagnosis of peripheral neuropathy irrespective of the cause.

## Supporting information

S1 Data (SAV)
